# Which Potential Linguistic Challenges do Pre-Service Teachers Identify in a Mathematical Expository Text?

**DOI:** 10.1007/s13138-023-00220-6

**Published:** 2023-04-26

**Authors:** Anselm R. Strohmaier, Isabel Albrecht, Anke Schmitz, Poldi Kuhl, Dominik Leiss

**Affiliations:** 1 Center for Empirical Research on Language and Education, Leuphana Universität Lüneburg, Lüneburg, Germany; 2 TUM School of Social Sciences and Technology, Technische Universität München, Arcisstr. 21, 80333 München, Germany; 3 Institut für Mathematik und ihre Didaktik, Leuphana Universität Lüneburg, Lüneburg, Germany; 4 Deutschdidaktik und ihre Disziplinen, PH Fachhochschule Nordwestschweiz, Windisch, Schweiz; 5 Institut für Bildungswissenschaft, Leuphana Universität Lüneburg, Lüneburg, Germany

**Keywords:** Language, Linguistically responsive teaching, Expository text, Explanation, Teacher competence, Linguistic challenges, Sprache, Sprachsensibler Mathematikunterricht, Erklärtext, Lehrkräftekompetenz, Sprachliche Hürden

## Abstract

Language is a crucial aspect of mathematical thinking and learning, and it is therefore essential for teachers to be equipped with the skills required for linguistically responsive teaching. This includes the ability to identify potential linguistic challenges in expository texts. In the present study, we investigated the ability of pre-service teachers (*N* = 115) to identify potential linguistic challenges in a mathematical expository text for ninth graders. Participants identified about 12% of the potential linguistic challenges that were previously identified by a reference expert group. They identified challenges more frequently that were on the word level and considered to be mathematics-specific by the experts. Subjective judgements of disciplinarity of the challenges differed between participants, and between participants and experts. No differences in the ability to identify potential linguistic challenges were found between participants who studied language arts (German or English) or mathematics as a subject. Our results indicate that pre-service teachers may not be adequately prepared to identify and respond to linguistic challenges in mathematical expository texts.

## Abstract (de)

Sprache ist ein wesentlicher Bestandteil mathematischen Denkens und Lernens, daher sollten Lehrkräfte in der Lage sein, Mathematikunterricht sprachsensibel zu gestalten. Dazu gehört auch die Fähigkeit, sprachliche Herausforderungen in mathematischen Erklärtexten zu identifizieren. In der vorliegenden Studie untersuchten wir die Fähigkeit von angehenden Lehrkräften (*N* = 115), potenzielle sprachliche Herausforderungen in einem mathematischen Erklärtext für Neuntklässler*innen zu identifizieren, und wie sie diese potenziellen Herausforderungen subjektiv als mathematikspezifisch oder fächerübergreifend einstuften. Die angehenden Lehrkräfte identifizierten etwa 12 % der potenziellen sprachlichen Herausforderungen, die von Expert*innen einer Referenzgruppe identifiziert wurden. Sie nannten dabei häufiger sprachliche Herausforderungen, die auf der Wortebene angesiedelt waren und von den Expert*innen als mathematikspezifisch angesehen wurden. Subjektive Beurteilungen der Disziplinarität der Aufgaben unterschieden sich zwischen den Teilnehmer*innen sowie zwischen Teilnehmer*innen und Expert*innen. Es wurden keine Unterschiede bezüglich der Fähigkeit, potenzielle sprachliche Herausforderungen zu identifizieren zwischen den Teilnehmer*innen festgestellt, die Sprachwissenschaften (Deutsch oder Englisch) oder Mathematik als Unterrichtsfach studierten. Unsere Ergebnisse deuten darauf hin, dass angehende Lehrkräfte möglicherweise nicht ausreichend auf den Umgang mit sprachlichen Herausforderungen in mathematischen Erklärtexten vorbereitet sind.

## Introduction

Language and mathematical performance are closely related in academic contexts (e.g., Abedi 2006; Peng et al. 2020; Pimm 1987; Prediger et al. 2018; Schleppegrell 2007, 2010) and language skills can influence future achievement in mathematics (e.g., Bailey et al., 2020; Duncan et al. 2007; Geary 2011; Gnambs and Lockl 2022; Paetsch and Kempert 2022; Paetsch et al. 2016; Ufer and Bochnik 2020; Ufer et al. 2013). Consequently, research and teaching practice should take the role of language for learning mathematics into account. In order to do this effectively, for instance through the implementation of *linguistically responsive mathematics teaching* (Lucas et al. 2008), teachers should be able to identify potential linguistic challenges and learning opportunities that may arise in mathematics classrooms, both in oral discourse and written texts and tasks (Bunch 2013). For example, textbooks containing expository texts are a typical resource in mathematics education (Fan et al. 2013) and it is important for teachers to be able to recognize linguistic challenges that students may encounter when reading such texts. However, no research has yet addressed teachers’ and pre-service teachers’ ability to identify potential linguistic challenges in mathematical expository texts.

In the present study, we addressed this ability, conceptualizing it as an important aspect of teachers’ expertise for language-responsive mathematics teaching. We also distinguished between potential linguistic challenges on the word level and the sentence level, and asked participants to subjectively categorize potential linguistic challenges as either mathematics-specific or cross-disciplinary. We then compared participants’ categorizations to those made by an expert group. Finally, we compared the ability to identify potential linguistic challenges of pre-service teachers with and without a language arts subject (German or English) and mathematics.

### Language and Mathematics Education

Language plays a dual role in mathematics education, serving as both a means of communication and a tool for thinking and learning (Pimm 1987; Vygotsky 1962). It plays an integral part in cognitive processes involved in learning mathematics, including complex conceptual understanding (Erath et al. 2021; Prediger and Şahin-Gür 2020; Ufer et al. 2020, 2013). Longitudinal studies showed that early language skills predict learning gains in mathematics in primary school (e.g., Bailey et al. 2020; Duncan et al. 2007; Lin and Powell 2021; Paetsch and Kempert 2022; Paetsch et al. 2016; Ufer and Bochnik 2020; Ufer et al. 2013; Vukovic and Lesaux 2013); while the causal links seem to get weaker in secondary school (Gnambs and Lockl 2022). As a result, interventions that target language skills can have a positive effect on learning mathematics (for an overview see Erath et al. 2021; see also Leiss and Plath 2020).

One aspect of mathematical thinking and learning in which language is important is the comprehension of mathematical texts. In mathematics classrooms, different kinds of written texts are common, including *expository texts* (Fan et al. 2013; Moschkovich 2013). Expository texts are texts that communicate information with the purpose for the reader to learn something (Weaver and Kintsch 1991). They are therefore defined by their functional goal (i.e., learning) and can differ in their rhetorical structure. Expository texts can be merely informative or persuasive (e.g., *refutational texts*; Tippett 2010). Here, we refer to informative expository texts, which Tippett calls “traditional” expository texts (2010, p. 958).

Reading mathematical expository texts can be an essential part of building mathematical knowledge and understanding (Fan et al. 2013; Österholm 2006). However, the language of mathematical expository texts can be challenging for some students, and specific linguistic features can hinder comprehension and learning (Schleppegrell 2007; van den Broek 2010). We refer to such features as *potential linguistic challenges*. This can include various language features on different levels and influence readers in different ways. Here, we focus on linguistic challenges of academic language.

### Potential Linguistic Challenges in Mathematical Expository Texts

The term *academic language *(Schleppegrell 2007; Snow & Ucelli 2009) refers to the features of language in school, which also covers the specific features of academic expository texts. Academic language and similar concepts have been discussed widely in research, but there is no single conceptual framework or universally accepted definition (see Snow and Ucelli 2009, for a discussion of existing definitions and conceptualizations). For example, academic language is closely related to the concept of *cognitive academic language proficiency* (CALP; Cummins 1979).

Academic language includes a variety of features, many of which can pose challenges for readers (Snow and Ucelli 2009). Within the present study, we characterized potential linguistic challenges in two common dimensions of academic language: their *disciplinarity* and their *linguistic level*. In the following sections, we will discuss each of these dimensions in more detail. In Sect. 1.3, we will then outline why this distinction is relevant when investigating teachers’ ability to identify potential linguistic challenges in mathematical expository texts.

#### Disciplinarity of Potential Linguistic Challenges

Some features of academic language are specific to a discipline (e.g., mathematics), while others can be considered cross-disciplinary and are common across subjects. For example, the use of variables is regarded as a unique way of displaying continuous meaning in mathematical language, while passive voice is often used in mathematics expository texts, but also in other disciplines (Österholm and Bergqvist 2013; Schleppegrell 2010; Zwiers 2014).

We refer to the features of academic language that are predominantly used in the context of one discipline as *disciplinary academic language*. It is also referred to as *content-area language* (Zwiers 2014), *discipline-specific language* (Uccelli et al. 2015; Zwiers 2014), or *subject-specific language *(Ufer and Bochnik 2020). Most students and teachers will come into contact and use these features of academic language predominantly at school (or in their teacher education program), but not outside of the disciplinary context. In the present study, we refer to the disciplinary academic language of mathematics, which we call *mathematics-specific academic language.*

Conversely, we refer to the features of academic language that are commonly used across disciplines as *cross-disciplinary academic language*. These features of academic language are used in a broader academic context. Therefore, students and teachers come into contact and use them across various situations at school and university. Students from households with higher educational backgrounds are typically also exposed to cross-disciplinary academic language at home (Snow and Uccelli 2009).

Even within disciplinary academic language, differences exist depending on the text genre, as well as between subdomains and content areas (Gottlieb and Ernst-Slavit 2013). For example, typical linguistic features of word problems might differ from those of mathematical proofs. Similarly, geometry has some unique linguistic features that are different from other content areas like statistics (Schleppegrell 2007, 2010). Thus, an investigation of academic language always has to consider the specific context including the discipline, content area, and text genre, and findings from one context might differ from another. Because the distinction between disciplinary and cross-disciplinary academic language is subjective and may depend on contextual and individual differences, it is not always clear-cut which linguistic features belong to which category (Zwiers 2014). Therefore, we consider this categorization to be a subjective judgement.

#### Linguistic Level of Potential Linguistic Challenges

Besides disciplinarity, academic language features are commonly classified by their linguistic level, including the *word level* and the *sentence level*. In addition, the *text level *or *discourse level* is often considered, with slightly different conceptualizations (Gottlieb and Ernst-Slavit 2013, Jost et al. 2017). The word level includes features of the academic lexis such as individual words or short phrases. The sentence level refers to characteristics of sentences structures such as word order or subordinate clauses. The text or discourse level describes features of academic language such as the organization and structure of texts, for instance, by cohesive devices across sentences or reference structures (Gottlieb and Ernst-Slavit 2013). While the word and sentence level can be distinguished quite clearly, the text or discourse level typically is intertwined with the other two (Prediger 2019). For example, coherence is considered a typical feature on the discourse level (Gottlieb and Ernst-Slavit 2013), but it can be increased by using fewer synonyms and pronouns on the word level or by adding connectives on the sentence level (McNamara and Magliano 2009). Because the discourse or text level is often not clearly separable from the other two levels, we only distinguished between features on the word and sentence level in the present study.

#### Examples for Potential Linguistic Challenges in Mathematical Expository Texts

With the distinction between cross-disciplinary and mathematics-specific features of academic language on the one hand and the distinction between the word level and the sentence level on the other hand, the potential linguistic challenges of academic language in mathematical expository texts can be characterized in a simplified 2 × 2 grid (see Table 1). The examples given here are illustrations of the categories as categorized by an expert group and based on prior research (see Sect. 2.3.3; Schleppegrell 2001, 2007, 2010; Snow and Uccelli 2009; Zwiers 2014). Note that the categorization of disciplinarity is considered subjective and might differ from person to person, while the categorization of the linguistic level is objective.

**Table 1:** Examples of Mathematics-Specific and Cross-Disciplinary Academic Language Features on the Word Level and the Sentence Level

	Cross-disciplinary	Mathematics-specific
Word level	Infrequent or complex vocabulary (e.g., *exemplary*) Idioms of academic language (e.g., *to make a point*)	Mathematical vocabulary (e.g., *equilateral*) Disciplinary idioms (e.g., *to isolate a variable)*
Sentence level	Passive voice Syntactical complexity (e.g., subordinate sentences, long sentences, complex structure) Morphosyntactic complexity (e.g., genitives, separable verbs, nominal phrases) Infrequent connectors (e.g., *consequently*) Referential complexity (e.g., pronouns, synonyms)	Mathematics-specific sentence constructions (e.g., *if-then*-statement) Referential complexity (e.g., use of variables, hierarchical mathematical terms)

At the word level, cross-disciplinary academic language typically includes infrequent vocabulary that is uncommon in everyday communication (Schleppegrell 2001). Its lexical inventory is characterized by high variability, where one concept may be described by similar words or synonyms with subtle differences (Snow and Uccelli 2009). Idioms represent a special form of lexical vocabulary. They are combinations of words that occur frequently and typically in combination with each other, thus forming a new lexical unit and a context-specific meaning (e.g., *to make a point*) (Zwiers 2007).

For mathematics-specific academic language, the word level is characterized by high density, precision, and technical terms (Schleppegrell 2007). Mathematical vocabulary may include words that are uniquely used in mathematics (e.g., *rectangular*), or words whose meaning differs from everyday language use (e.g., *product*; Schleppegrell 2007). Mathematical lexis includes specific idioms that typically describe mathematical actions such as *solving an equation, constructing a figure*, or *isolate a variable* (Zwiers 2014).

Categorizing linguistic features on the word level can be subjective and context-dependent. For instance, the word “proof” can be either a specific mathematical concept or part of a broader cross-disciplinary academic language, depending on the context and the reader’s subjective interpretation.

The sentence level of cross-disciplinary academic language typically includes specific syntactic, grammatical, and referential features. Complex syntax is a typical feature of academic language, with passive voice, long subordinate clauses, insertions, and a sentence structure that differs from the usual subject-predicate-object structure (Schleppegrell 2001; Zwiers 2014). Furthermore, complex nominal phrases, separable verbs and genitive constructions contribute to high information density (Schleppegrell 2001; Snow and Uccelli 2009; Zwiers 2014). Additionally, academic language is characterized by the use of infrequent connectors (e.g., *consequently, thereby, insofar as*) and less transparent references created by the use of pronouns instead of nouns and synonyms instead of noun repetition.

The sentence level of mathematical language is often characterized by conventions about typical modes of expression (Schleppegrell 2007). This includes specific grammatic patterns of mathematical definitions or theorems. For example, the *if-then-*statement has a unique meaning in mathematics by establishing explicit logical relationships between statements, which differs from its use in everyday language. The concept of variables and the use of hierarchical concepts (e.g., for quadrilaterals) lead to a mathematics-specific form of referential complexity: When variables are used, the reader needs to constantly keep information about the underlying value or object in their working memory, conceptually similar to the use of pronouns. With hierarchical concepts, different terms might be used which refer to the same properties, similar to the use of synonyms (e.g., “Rectangles have four right angles, so the sum of angles in a square must be 360°”).

Like on the word level, some linguistic features on the sentence level may be associated with a specific discipline, but for others, the categorization can be ambiguous. For example, long noun phrases are often used in mathematical proofs and might be considered characteristic of mathematics-specific language (Schleppegrell 2007). However, they may also appear in other types of texts and disciplines.

### Teachers’ Ability to Identify Potential Linguistic Challenges in Mathematical Expository Texts

As a result of the increased awareness of the role of language in mathematics education and other subjects, research has argued that teachers need to identify and respond to the role of language in their classrooms (e.g., Wessel and Erath 2018; Zwiers 2014). This need can be addressed through *linguistically responsive teaching *(Lucas et al. 2008). It addresses the challenges and potential of incorporating linguistic perspectives in all subjects, not only in the language arts. This also applies to the work with written texts (Bunch 2013). In the following, we review frameworks of general teacher expertise and expertise for linguistically responsive mathematics teaching and how they relate to the work with mathematical expository texts. We argue that the *ability to identify potential linguistic challenges* is reflected in all of these frameworks. We conclude that this ability is necessary for working with mathematical expository texts in a linguistically responsive way. Importantly, this does not imply that teachers also need to be able to categorize these challenges regarding their disciplinarity and linguistic level according to experts. However, knowing the linguistic level of these identified challenges and the subjective judgement regarding their disciplinarity is a valuable characterization of this ability.

#### The Concept of Identifying Potential Linguistic Challenges in Frameworks of Teacher Expertise

The ability to identify potential linguistic challenges is reflected in many of the most popular frameworks of language-related abilities for teachers. Fillmore and Snow (2002) argue that teachers of all subjects need *educational linguistics *to account for the role of language in their classrooms. This is required to fulfill a total of five language-related functions, of which the first two require the ability to identify potential linguistic challenges: The teacher as *communicator* has the ability to identify an adequate language (and thus, potential challenges) for teaching. The teacher as *educator* needs to select materials and activities to teach effectively, which requires knowledge about language in connection with the content and the ability to identify cognitive obstacles to learning caused by linguistic challenges. The remaining three functions that do not explicitly refer to the identification of potential challenges are the teacher as *evaluator* (able to make valid judgements in the presence of language behavior), as *educated human being* (with basic knowledge about language) and as *agent of socialization *(aware of their role as a representative of the social world outside of the home).

Galguera (2011) and Bunch (2013) introduced the concept of *pedagogical language knowledge* (PLK) to describe teachers’ language knowledge and linguistic proficiency as a facet of teachers’ professional knowledge. They situate PLK as an addition to the knowledge categories by Shulman (1986). Pedagogical language knowledge refers to “knowledge of language directly related to disciplinary teaching and learning and situated in the particular (and multiple) contexts in which teaching and learning take place” (Bunch 2013, p. 307). Bunch (2013) argues that PLK includes knowledge of the linguistic features of disciplinary texts and tasks, as well as the ability to identify and address potential linguistic challenges.

Lucas and Villegas (2013) and Lucas et al. (2008) assume that the required expertise for linguistically responsive teaching comprises *orientations* and *pedagogical knowledge and skills*. Orientations include *sociolinguistic consciousness, value for linguistic diversity*, and an *inclination to advocate for English language learners*. Pedagogical knowledge and skills include strategies for learning about the linguistic and academic background of learners and for scaffolding instruction, and the ability to apply principles of second language learning. Importantly, it also involves to the *ability to identify the language demands of classroom tasks*.

The ability to identify potential linguistic challenges can also be found in models of professional teacher competence without a specific focus on language: Being able to judge the potential of tasks for learning, referred to as *task diagnosis*, is part of conceptualizations of teacher competence (e.g., in the DiaCoM framework, Loibl et al. 2020; see also Bromme 1981; Rieu et al. 2022; Schreiter et al. 2021; Smith and Stein 1998, or in conceptualizations of PCK, e.g., Binder et al. 2018). Task diagnosis forms the basis for the selection of tasks that are appropriate and purposeful for the learning goals and students’ learning prerequisites (Karst et al. 2017; Rieu et al. 2022). It includes identifying potentially relevant task features and difficulties (Philipp 2018; Rieu et al. 2022) and therefore, should also extend to linguistic challenges. Typically, studies on teachers’ task diagnosis focus on estimating the overall task difficulty and not individual difficulty generating factors. In particular, to our knowledge, no studies from these research traditions have focused on the identification of linguistic task features.

References to identifying potential language challenges can also be found in other frameworks of professional teacher competence, e.g., “knowledge […] of tasks, their cognitive demands and the prior knowledge they implicitly require*”* in COACTIV (Baumert and Kunter 2013, p. 2; see also Binder et al. 2018) or “using mathematical language and critiquing its use” in the MKT framework (Ball et al. 2008, p. 400). Notably, in these models of professional teacher competence, these abilities and knowledge facets are considered a part of PCK, while the concept of PLK as described by Bunch (2013) and Galguera (2011), is seen as distinct from PCK.

Specific to mathematics teaching, Prediger (2019) distinguishes *jobs, practices, pedagogical tools, orientations* and *categories *as aspects of linguistically responsive teaching. Jobs refer to the situational demands of subject-matter teaching, pedagogical tools are applied to coping with the job, orientations refer to beliefs that guide the handling of the jobs, and categories are conceptual knowledge elements that structure the thinking of the teacher with regard to these processes. Similar to the models above, she argues that the job of identifying mathematically relevant language demands has a superordinate role in linguistically responsive mathematics teaching, as it is the prerequisite for further purposeful and sufficient teacher action (see also Moschkovich 2013).

#### Prior Research on Teachers’ Ability to Identify Potential Linguistic Challenges

Only few studies have empirically studied the linguistic expertise of pre-service teachers for linguistically responsive teaching, and even fewer studies have focused on mathematics education or the ability to identify potential linguistic challenges. Additionally, the majority of these studies have focused on classroom interaction rather than on expository texts. In an observational study with three teachers of science, language arts, and social studies, Zwiers (2007) found that teachers who were able to identify language features that were important for disciplinary learning were likely to be better prepared for linguistically responsive teaching. Wallner (2021) reported that their sample of 95 teachers were primarily sensitive to possibly challenging language features on a surface level, but not on a deeper, conceptual level. In a study with 322 teachers of varying expertise and subject, Neugebauer and Heineke (2020) found that the majority had substantial deficits in terms of a holistic understanding of relevant linguistic challenges for teaching and learning in a specific discipline.

Typically, studies focusing on mathematics analyzed teacher practices of successful or unsuccessful teachers in mathematics classrooms or teacher development programs descriptively (e.g., Adler 1995; Essien et al. 2016; Prediger 2019; Turner et al. 2019), identifying successful practices for linguistically responsive mathematics teaching. However, to our knowledge, only two studies investigated whether teachers have the necessary expertise regarding potential linguistic challenges to engage in linguistically responsive mathematics teaching: Jost et al. (2017) compared trained language coaches to ten regular mathematics teachers and found that the language coaches had a much broader and more nuanced perspective of potential linguistic challenges as a basis for linguistically responsive teaching. Prediger et al. (2019) investigated which categories 223 secondary education mathematics teachers focused on when analyzing students’ written explanations. They reported that while there was a wide range of possible categories for analyzing language features, few referred to the level of conceptual understanding.

Overall, it appears that teachers, including mathematics teachers, rarely identify linguistic features which are potentially challenging for students and relevant for linguistically responsive teaching, but only few quantitative results exist to support this. Accordingly, little is known about the nature of the deficits in the ability to identify potential linguistic challenges and about ways to address them, particularly from a mathematics education perspective. Moreover, to our knowledge, no previous study investigated teacher expertise with regard to mathematical expository texts.

#### Differences in Identifying Potential Linguistic Challenges by Linguistic Level and Disciplinarity

In order to work with mathematical expository texts in a linguistically responsive way, pre-service teachers should ideally be able to identify all potential linguistic challenges, not just those specific to a discipline or on one linguistic level. Investigating if differences regarding these dimensions exist can help to better understand the ability to identify potential linguistic challenges. This, in turn, may suggest different consequences.

Whether pre-service teachers are able to identify potential linguistic challenges more frequently if they are considered mathematics-specific or cross-disciplinary may influence how well they are prepared for linguistically responsive teaching with mathematical expository texts. Both facets of academic language are relevant, but teachers might focus primarily on linguistic features that they consider mathematics-specific when identifying potential difficulties in a mathematical expository text. Such a lack of attention to cross-disciplinary academic language could be problematic for several reasons. For example, because school students from households with higher educational backgrounds are typically exposed to cross-disciplinary academic language earlier and more frequently, they tend to have fewer problems with it (Snow and Uccelli 2009). Accordingly, they can then focus their resources on acquiring disciplinary academic language, while students who use academic language predominantly at school need more time and resources to get accustomed to cross-disciplinary academic language (Zwiers 2007). If pre-service teachers tend to focus solely on mathematics-specific linguistic challenges, this might disadvantage students from lower educational backgrounds.

The distinction between mathematics-specific linguistic challenges and cross-disciplinary linguistic challenges is subjective and context-dependent, with possibly differing interpretations among students, teachers, and experts. This subjectivity raises important questions about the consistency of pre-service teachers’ categorization of linguistic challenges and how well these align with expert perspectives. Lack of consistency in categorization could impact teaching in several ways. If pre-service teachers have varying interpretations of the disciplinarity of linguistic challenges, this could affect their assessment of the difficulty of expository texts for students and their interpretation of students’ difficulties. Moreover, if teachers and experts do not have a common understanding of the disciplinarity of linguistic challenges, it becomes more difficult for teachers to apply research findings to their practice. Therefore, it would be valuable to investigate and establish a common understanding of the disciplinarity of linguistic challenges among pre-service teachers and experts, particularly if there is a lack of consensus among teachers and experts regarding the categorization of linguistic challenges. The question how this relates to the disciplinarity from the students’ perspective would be an important next step, but is not addressed in the present study.

Regarding linguistic levels, linguistic features on the word and sentence level both contribute to a deep and conceptual understanding, including in mathematical expository texts (Moschkovich 2013; Pimm 1987; Schleppegrell 2007, 2010; Uccelli et al. 2015). However, potential linguistic challenges at the word level are typically easier to identify for teachers (Prediger 2019; Wallner 2021). Moreover, a common misconception about mathematics-specific academic language is that it consists mainly of specific vocabulary (Zwiers 2007, 2014). For example, the findings by Jost et al. (2017) indicate that teachers without specific training rarely consider linguistic features on the sentence level. However, it is unclear if this deficit is reflected in pre-service teachers regarding mathematical expository texts. If teachers identify potential linguistic difficulties primarily at the word level, instruction may become fixated on these features and not provide helpful support for learners who have comprehension problems at the sentence level.

Pre-service teachers’ ability to identify linguistic challenges may vary based on their subject of study. For example, language arts teachers may be more attuned to academic language features which are categorized to be cross-disciplinary by experts, while mathematics teachers may focus more on mathematics-specific language features. This difference could be due to differential exposure during training and practice, subject choice based on beliefs or prior knowledge, or differences in teacher education programs (Brandt et al. 2023). However, it is also possible that pre-service teachers’ subject does not play a role, since potential linguistic challenges for students might be so obvious that subject-specific knowledge is not relevant, or since all teachers may have encountered mathematical language during their own schooling.

### The Present Study

The ability to identify potential linguistic challenges is considered a requirement for linguistically responsive teaching when working with mathematical expository texts. In the present study, we investigated how many potential linguistic challenges pre-service teachers identified in an expository text, compared to an expert group consisting of linguistics and mathematics educators. In a second step, we analyzed if these identified challenges were on the word level or the sentence level, and how they were subjectively categorized either as mathematics-specific or cross-disciplinary by the pre-service teachers themselves. Furthermore, we used an expert groups’ categorization to compare the amount of identified potential difficulties regarding disciplinarity and linguistic level. Finally, we investigated differences depending on pre-service teachers’ subjects. We posed the following research questions:

#### RQ 1:

How many potential linguistic challenges that were previously identified by an expert reference group do pre-service teachers identify in a mathematical expository text?

#### RQ2:

Of these identified potential linguistic challenges, which do pre-service teachers consider to be mathematics-specific, and which to be cross-disciplinary?

#### RQ3:

Based on the experts’ categorization, are linguistic challenges identified more often if they are mathematics-specific or cross-disciplinary? Are they identified more often if they are on the word level or on the sentence level?

#### RQ4:

Does the ability to identify potential linguistic challenges differ depending on pre-service teachers’ subjects (German or English language arts vs. mathematics)?

Based on previous research, we expected (1) that pre-service teachers would be able to identify only few of the potential linguistic challenges, and (2) that they should agree on the judgement of disciplinarity in most, but not all cases. We expected (3) that pre-service teachers might show a tendency towards identifying more potential linguistic challenges on the word level than on the sentence level. We further expected (4) differences in the identified potential linguistic challenges based on pre-service teachers’ subjects: We expected that pre-service teachers with German or English language as a subject would identify more potential linguistic challenges that were considered to be cross-disciplinary by experts, while pre-service teachers with mathematics as a subject would predominantly identify mathematics-specific potential linguistic challenges. Because the categorization of disciplinarity can differ subjectively, the analyses of RQ3 and RQ4 were based on the categorization of the expert group to provide a consistent reference across participants. For these questions, it was ignored how the pre-service teachers themselves had categorized the potential linguistic challenges in terms of disciplinarity for RQ2.

## Methods

### Sample

*N* = 135 students from a pre-service teacher education program participated in the study by answering an online questionnaire. 18 participants (13%) were excluded because they started but did not complete the questionnaire. Two more participants were excluded due to meaningless response patterns. The remaining participants were *n* = 115 pre-service teachers (5 male, 110 female). They were, on average, in the seventh semester of the teacher education program (*M* = 7.13, *SD* = 2.46). 84 participants were between 21 and 25 years old, 18 participants were between 26 and 30 years old, 2 participants were younger, and 11 participants were older.

Participants were students in primary and secondary teacher education programs for various school subjects. At the beginning of their studies, they had been required to choose two subjects out of 11 subjects, which could result in almost any combination of the school subjects taught in the German school system. Therefore, participants in our study could either be pre-service teachers of a combination of German or English language arts *and* mathematics, one of the three in combination with another subject (e.g., art), or neither (e.g., if they chose art and biology). Table 2 gives the number of pre-service teachers with or without a language arts subject and with or without mathematics. It shows that the majority of pre-service teachers studied mathematics without studying German or English language arts (*n* = 63). Combinations of mathematics and German or English language arts were scarce (*n* = 10).

**Table 2:** Distribution of Participants by Subject

		Mathematics		
		Yes	No	Total
German or English language arts	Yes	10	63	73
	No	34	8	42
Total		44	71	115

*Language in mathematics education* was not an explicit topic of the curriculum of any participant prior to the assessment, and the mathematics pre-service teachers at the time of the survey had only received basic subject-specific and subject-didactic courses in arithmetic, geometry, and functions.

All participants completed the survey on a voluntary basis and provided informed consent through a checkbox at the beginning of the questionnaire. Participants were informed about the goal, the procedure and the expected duration of the study, as well as the data handling. The debriefing included a thank you and contact information. The study was conducted in accordance with the 2017 *Ethical Principles of Psychologists and Code of Conduct of the American Psychological Association*. Ethics approval was not required by institutional guidelines or national regulations.

### Procedure

**Fig. 1:**
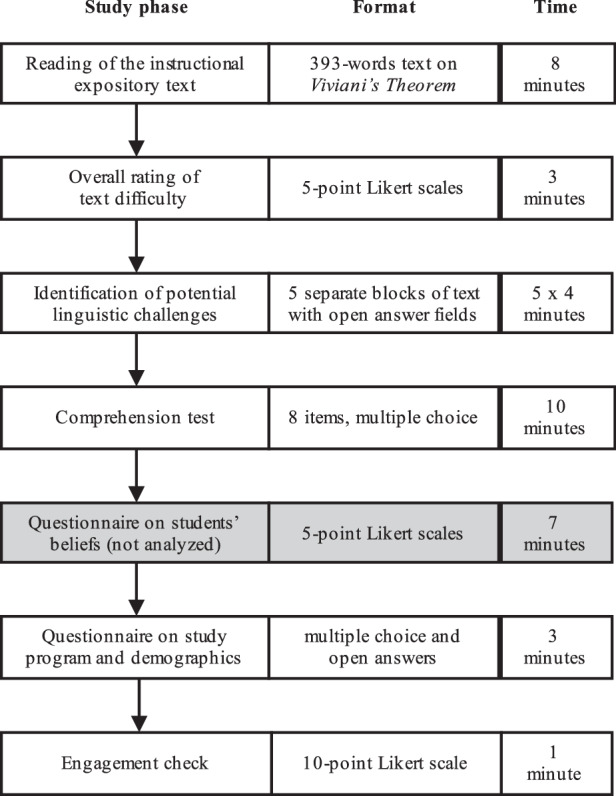
Illustration of the study phases

Participants completed the online questionnaire during a course on either methodology (5th semester) or school achievement (9th semester) which were taught online due to the COVID-19 pandemic. An overview of the complete procedure is given in Fig. 1.

After giving informed consent for participation, participants were presented with an expository text (see 2.3.1). They read the text for comprehension, and were then asked to rate the overall cross-disciplinary and mathematics-specific linguistic difficulty of the text as well as the difficulty of the content (see 2.3.2). In the next step, participants were presented with single paragraphs of the text and were asked to identify and list potential linguistic challenges in each paragraph, and categorize them either as cross-disciplinary or mathematics-specific (see 2.3.3). Finally, participants completed a comprehension test (see 2.3.4), a background questionnaire, an engagement check (see 2.3.5), and were then automatically thanked and debriefed. There was no time limit on the assessment, but estimates are given in Fig. 1.

### Materials and Instruments

#### Expository Text

The expository text was a 393-words explanation about *Viviani’s Theorem*. The theorem states that the sum of the distances from any interior point of an equilateral triangle to its sides equals the length of the triangle’s altitude. An example paragraph is given in Appendix A. The text was designed to reflect an age-appropriate challenge for 9th-graders regarding linguistic and mathematical difficulty. The text falls under the definition of a traditional expository text by Weaver and Kintsch (1991) and Tippett (2010), as it was an informative text with the goal of learning. The Flesch reading ease score was* FREgerman* = 61.6, which is considered *easy *and appropriate for 9th-graders (“einfach”; Amstad 1978; Flesch 1948)^1^.

#### Overall Rating of Text Difficulty

The rating of the overall cross-disciplinary and mathematics-specific linguistic difficulty of the text as well as the difficulty of the mathematical content for 9th grade students was assessed with three 5‑point Likert scales (“Diese Erklärung ist für einen Schüler/eine Schülerin der 9. Klasse einer Oberschule [sprachlich/fachsprachlich/inhaltlich] …” [“For a student in 9th grade of secondary school, this explanation is [linguistically/disciplinary-linguistically/with regard to the content] …”]; 1 = “not difficult”, 5 = “very difficult”).

#### Identification of Potential Linguistic Challenges

To assess participants’ ability to identify potential linguistic challenges, five single paragraphs were taken from the expository text that were between 31 and 54 words long and, in combination, of similar difficulty as the whole text, *FREgerman* = 62.5. Each paragraph was presented on one page with two separate open answer fields on the same page where participants were asked to list everything that might reflect linguistic challenges for 9th grade students, either cross-disciplinary or mathematics-specific (“Bitte benennen Sie alles, was für einen Schüler/eine Schülerin der 9. Klasse einer Oberschule in diesem Erklärungsausschnitt [fachsprachlich/sprachlich] schwierig ist. Belegen Sie alle identifizierten Schwierigkeiten mit Beispielen aus dem Text.” [“Please list anything that is [disciplinary linguistically/linguistically] difficult for a 9th grade high school student in this explanation. Provide examples from the text for each identified challenge”]). Prior to the experiment, language experts and mathematics education researchers^2^ identified a total of 44 potential linguistic challenges in these five paragraphs (see Appendix A for an example). These potential linguistic challenges were further categorized as being either on the word level or the text and sentence level by the expert group (see Table 3).

**Table 3:** Number of Potential Linguistic Challenges Identified by the Expert Group

Level	Mathematics-specific	Cross-disciplinary	Overall
Word	13 (e.g., *equilateral*)	9 (e.g., *arbitrary*)	22
Sentence	8 (e.g., if-then statement)	14 (e.g., genitive *the program’s*)	22
Overall	21	23	44

#### Comprehension Test

To check whether participants were able to understand the mathematical content of the expository text, comprehension was evaluated with a single-choice format test at the end of the experiment, including recall and transfer questions (8 items, Cronbach’s *α* = 0.71; e.g., “Welche der folgenden Aussagen gilt für dieses Dreieck?” [“Which of the following statements is true for this triangle?”]).

#### Background Questionnaire, Demographic Data, and Engagement Check

At the end of the online questionnaire, participants were asked about basic demographic data (e.g., age, gender), about their studies (e.g., semester, subject) and about prior knowledge (e.g., school degree grades). Participants’ engagement was assessed with one item on a 10-point Likert scale (“Wie sehr haben Sie sich bei der Bearbeitung dieser Befragung bemüht?” [How much effort did you put into completing this survey?], 1 = “not at all”, 10 = “very much”).

### Data Analysis

Participants’ responses in the identification task were independently double-coded by two research assistants, one a linguist and one a mathematics education researcher (both were also part of the expert group which previously identified potential linguistic challenges). The coders checked which of the 44 potential linguistic challenges previously identified in the expert rating were also identified by the participants^3^, dichotomously rating each potential challenge as “identified” or “not identified”. For this rating, it was irrelevant if participants agreed with the expert group on the disciplinarity of the potential linguistic challenges. Thus, a potential linguistic challenge that the experts considered to be mathematics-specific would also count if it was named as cross-disciplinary, and vice versa^4^. Inter-rater reliability was excellent, Cohen’s *κ* = 0.90. Cases where coders disagreed were decided by a third, independent coder.

For RQ1, the total number of different identified potential linguistic challenges (regardless if it was categorized as cross-disciplinary or mathematics-specific) was calculated per participant.

For RQ2, we counted how often each potential linguistic challenge was named in the answer field for cross-disciplinary potential linguistic challenges and in the answer field for mathematics-specific challenges, respectively. The absolute values were divided by the number of participants, resulting for each potential linguistic challenge in the proportion of participants that had named it in each of the two categories.

For statistical analyses for RQ3 and RQ4, the percentage of identified potential linguistic challenges by participant was calculated separately for cross-disciplinary challenges on the word level, mathematics-specific challenges on the word level, cross-disciplinary challenges on the sentence level, and mathematics-specific challenges on the sentence level. For these analyses, it was ignored whether participants named a challenge in the answer field for mathematics-specific or cross-disciplinary challenges, and the categorization of the expert group was used for analyses. The percentage for each category was calculated by dividing the participant’s number of identified challenges by the number of challenges identified by the experts.

To investigate systematic differences in the percentage of identified potential linguistic challenges, we conducted a 2 × 2 within-subject ANOVA with the factors *disciplinarity* (mathematics-specific vs. cross-disciplinary) and *linguistic level* (word level vs. sentence level). For RQ4, we further included participants’ study subjects as 2 × 2 between-subject factors (mathematics vs. no mathematics; German or English language arts vs. no language subject).

In all analyses regarding pre-service teachers’ subject, the interaction of the between-subject factors *mathematics* and *language arts* was included in the models. However, due to the small number of participants with both or neither subject (see Table 2), these interactions were not meaningfully interpretable and are not reported here.

## Results

### Preliminary Analysis: Comprehension Test, Perceived Difficulty, Engagement

**Fig. 2:**
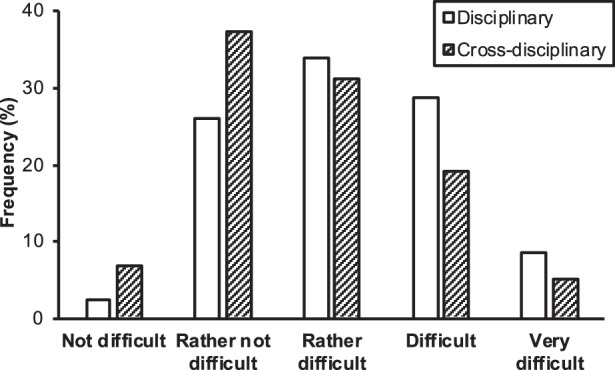
Frequency of participants’ responses regarding the perceived linguistic difficulty of the text for 9th grade students

The majority of participants perceived the expository text to be at least “rather difficult” for 9th graders (Fig. 2), 71.3% for mathematics-specific linguistic difficulty, 55.7% for cross-disciplinary linguistic difficulty). On average, the mathematics-specific linguistic difficulty was perceived higher, *M* = 3.15, *SD* = 0.99 than the cross-disciplinary linguistic difficulty, *M* = 2.78, *SD* = 1.01, *t*(114) = 4.55,* d* = 0.37, *p* < 0.001, 95% CI [0.20, 0.52], 1‑β = 0.98. No differences were found for this rating between pre-service teachers of different subjects, *F*(1, 111) = 2.71, *p* = 0.102, part. *η*^*2*^ = 0.02, 1‑β = 0.37 for mathematics, *F*(1, 111) = 1.69, *p* = 0.196, part. *η*^*2*^ = 0.02, 1‑β = 0.37 for language arts.

In the comprehension test, the mean solution rate of *M* = 77.2%, *SD* = 23.6% indicated that the mathematical content of the expository text was well understood. Pre-service mathematics teachers (*M* = 85.8%, *SD* = 19.7%) performed better than pre-service teachers without mathematics as a subject (*M* = 71.8%, *SD* = 24.3%), *F*(1, 111) = 6.94, *p* = 0.010, part. *η*^*2*^ = 0.06, 1‑β = 0.74. No effect was found regarding language arts subjects,* F*(1, 111) = 0.22, *p* = 0.643, part. *η*^*2*^ < 0.01, 1‑β = 0.08.

Participants reported a low content difficulty for 9th graders, *M* = 1.69, *SD* = 0.95. No main effects of pre-service teachers’ subjects were found, *F*(1, 111) = 3.11, *p* = 0.080, part. *η*^*2*^ < 0.03, 1‑β = 0.42 for mathematics, *F*(1, 111) = 0.97, *p* = 0.268, part. *η*^*2*^ = 0.01, 1‑β = 0.20 for language arts.

Participants reported a mean effort of *M* = 7.70, *SD* = 1.66 (1 = “none”, 10 = “very much”). No differences were found regarding the reported effort between pre-service teachers’ subjects, *F*(1, 111) = 0.00, *p* = 0.972, part. *η*^*2*^ < 0.01, 1‑β = 0.05 for mathematics, *F*(1, 111) = 0.45, *p* = 0.504, part. *η*^*2*^ < 0.01, 1‑β = 0.10 for language arts.

### RQ1: Overall Ability to Identify Potential Linguistic Challenges

Regarding RQ1 on pre-service teachers’ ability to identify potential linguistic challenges for 9th graders, the analyses showed that of the 44 potential linguistic challenges that had been identified by the expert group prior to the experiment, 40 were identified by the participants at least once. Participants identified a mean of *M* = 5.44, *SD* = 4.77 potential linguistic challenges, which corresponded to about one in eight (12.4%) challenges that were identified by the experts^5^. The maximum number of identified challenges was 23 (52.3%, 1 participant), the minimum was zero (0%, 16 participants)^6^.

**Fig. 3:**
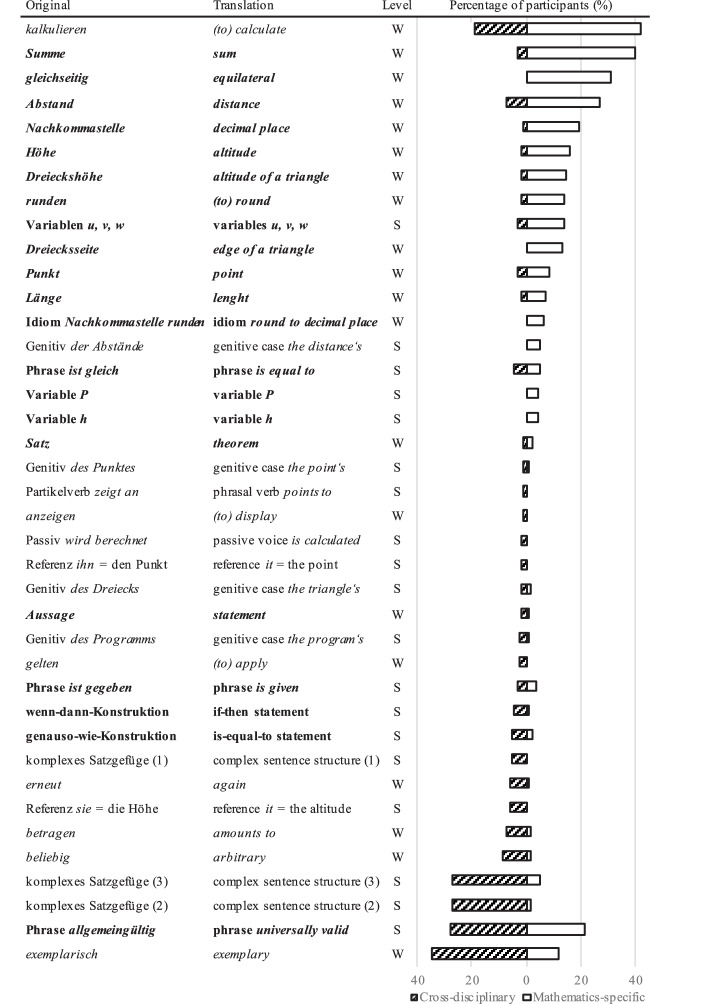
Percentage of participants identifying potential linguistic challenges as cross-disciplinary or mathematics-specific. Linguistic levels: W = word level, S = sentence level. The complex sentence structures are given in Appendix B. Potential linguistic challenges in bold had been categorized as mathematics-specific by the expert group

Figure 3 illustrates how often each individual potential linguistic challenge was identified. Only eight potential linguistic challenges were identified by more than 20% of the pre-service teachers, and the majority of potential challenges was only identified sporadically.

### RQ2: Cross-Disciplinary and Mathematics-Specific Potential Linguistic Challenges

Results for RQ2, which asked how pre-service teachers categorize the potential linguistic challenges as cross-disciplinary or mathematics-specific, are illustrated in Fig. 3. It shows the percentage of participants that categorized each linguistic difficulty as cross-disciplinary or mathematics-specific, sorted from potential linguistic challenges that were predominantly categorized as mathematics-specific (top) to challenges that were predominantly categorized as cross-disciplinary (bottom). The experts’ categorization is also indicated in Fig. 3. 9 of the 10 potential linguistic challenges which were categorized by participants as mathematics-specific with the highest frequency were on the word level, whereas this was only true for 4 of the 10 challenges which were categorized as cross-disciplinary most frequently. Table 3 shows that this tendency was also present in the expert rating, but less pronounced (62% of mathematics-specific potential challenges were on the word level, compared to 39% for cross-disciplinary potential linguistic challenges).

Moreover, it was obvious that the distinction of disciplinarity was not clear-cut across pre-service teachers. While some potential linguistic challenges were clearly identified as being either mathematics-specific or cross-disciplinary, there were also instances where participants did not agree. For example, while participants categorized the term *gleichseitig *[equilateral] only as a mathematics-specific potential linguistic challenge and a complex sentence structure only as cross-disciplinary, the phrase *allgemeingültig *[universally valid] was named in both categories (see Fig. 3). Thus, the distinction between mathematics-specific and cross-disciplinary language was inconsistent across participants. Similarly, pre-service teachers did not always agree with the expert group. For example, the experts categorized the term *kalkulieren *((to) calculate) as cross-disciplinary, whereas the majority of pre-service teachers categorized it as mathematics-specific.

### RQ3: Identified Potential Linguistic Challenges by Disciplinarity and Linguistic Level

Addressing RQ3, Table 4 shows the percentages of identified potential linguistic challenges by linguistic level and by the experts’ judgement of disciplinarity. To test for the significance of differences, a 2 (*linguistic level*: word vs. sentence) × 2 (*disciplinarity*: cross-disciplinary vs. mathematics-specific) within-subject ANOVA was performed. The analysis showed a significant main effect for *linguistic level, F*(1, 114) = 31.67, *p* < 0.001, part*. η*^*2*^ = 0.22, 1‑β = 1.00, such that potential linguistic challenges on the word level were identified more frequently than potential linguistic challenges on the sentence level. The main effect for *disciplinarity* was also significant, *F*(1, 114) = 11.75, *p* < 0.001, part. *η*^*2*^ = 0.09, 1‑β = 0.93, such that potential linguistic challenges that were categorized as mathematics-specific by the experts were identified more often than cross-disciplinary potential linguistic challenges. The interaction effect was significant, *F*(1, 114) = 6.16, *p* = 0.015, part. *η*^*2*^ = 0.05, 1‑β = 0.69: Potential linguistic challenges on the word level were identified about equally often, whereas potential linguistic challenges on the sentence level were identified more often if they were mathematics-specific.

**Table 4:** Percentages of Identified Potential Linguistic Challenges by Linguistic Level and Disciplinarity

Level	Mathematics-specific (%)	Cross-disciplinary (%)	Overall (%)
Word	16.52 *(20.26)*	15.17 *(14.59)*	15.97 *(15.84)*
Sentence	12.93 *(14.33)*	6.40 *(8.05)*	8.77 *(7.91)*
Overall	15.16 *(15.93)*	9.83 *(8.74)*	12.37 *(10.85)*

*Note. *Standard deviations are given in parentheses

### RQ4: Differences Between Pre-Service Teachers of Different Subjects

When the between-subject factors regarding participants’ subjects (mathematics vs. no mathematics; German or English language arts subject vs. no language arts subject) were added to the ANOVA, similar results emerged regarding the main effects of *linguistic level, disciplinarity*, and their interaction (*linguistic level*: *F*(1, 111) = 10.60, *p* = 0.001, part. *η*^*2*^ = 0.09, 1‑β = 0.90; *disciplinarity*: *F*(1, 111) = 4.26, *p* = 0.041, part. *η*^*2*^ = 0.04, 1‑β = 0.53; interaction: *F*(1, 111) = 2.98, *p* = 0.087, part. *η*^*2*^ = 0.03, 1‑β = 0.40). Neither the main effects of mathematics, *F*(1, 111) = 1.63, *p* = 0.205, part. *η*^*2*^ = 0.01, 1‑β = 0.24, or language arts subject, *F*(1, 111) = 2.74, *p* = 0.101, part. *η*^*2*^ = 0.02, 1‑β = 0.38, on the percentage of identified potential linguistic challenges were significant.

Additional separate ANOVAs where subjects were added individually were conducted. They showed similar, nonsignificant effects for mathematics, *F*(1, 113) = 0.014, *p* = 0.907, part. *η*^*2*^ = 0.00, 1‑β = 0.05, and language arts subject, *F*(1, 113) = 1.28, *p* = 0.261, part. *η*^*2*^ = 0.01, 1‑β = 0.20. None of the cross-level interaction effects (*subject* × *disciplinarity, subject* × *linguistic level*, and *subject* × *disciplinarity* × *linguistic level*) on the percentage of identified potential linguistic challenges were significant (*η*^*2*^ = 0.007, *p* > 0.374). Accordingly, we did not find evidence that the fact if participants studied German or English language arts or mathematics influenced how many or which potential linguistic challenges they identified. Post-hoc sensitivity power analyses (Cohen 1988) resulted in a minimal detectable effect size of *η*^*2*^ ≈ 0.06 for a statistical power (1-β) of 0.8 at α = 0.05 for the between-subject effects and between-between interaction effects in these ANOVAs.

## Discussion

Previous research agrees that language plays an important role in mathematics education and that teachers need to be equipped with a range of language-related abilities. Frameworks of these abilities (e.g., Bunch 2013; Fillmore and Snow 2002; Lucas and Villegas 2013; Lucas et al. 2008; Moschkovich 2013; Prediger 2019) emphasize that the ability to identify language demands is a prerequisite for informed and targeted actions towards linguistically responsive mathematics education. This includes teachers’ ability to identify potential linguistic challenges in expository texts (Bunch 2013). However, previous research in mathematics education has mostly used interviews or observational data to assess whether teachers identify potential linguistic challenges but no experimental assessment of their ability to identify these challenges (e.g., Adler 1995; Essien et al. 2016; Prediger 2019; Turner et al. 2019). Moreover, previous research has rarely distinguished between different facets of potential linguistic difficulties. This is necessary to understand how teachers are prepared for linguistically responsive teaching, and to derive consequences for teacher education. Moreover, most previous research focused on classroom interaction and communication, but not the work with expository texts, even though texts are a vital aspect of teaching mathematics (Fan et al. 2013; Moschkovich 2013). In the current study, we addressed these open questions.

### Pre-Service Teachers’ Ability to Identify Potential Linguistic Challenges

With regard to RQ1, our results show that the participating pre-service teachers identified only a relatively small number of potential linguistic challenges in a mathematical expository text when compared to the group of experts. These results extend previous research to the work with expository texts and by providing the experts’ rating as a baseline. Judging from the theoretical frameworks, this deficit might negatively affect pre-service teachers’ ability to work with expository texts in a linguistically responsive way, as the identification of challenges is considered a prerequisite for further targeted action (e.g., Prediger 2019).

Probably, not all of the 44 challenges identified by the expert group negatively affect comprehension or learning for all students at school. However, we argue that the decision which aspects of a text need to be accounted for first requires the identification of *potential* challenges even if they are considered irrelevant for this particular situation or student in a later stage. In our understanding, this is in line with models of linguistically responsive mathematics teaching that stress the superordinate role of being sensitive to potential linguistic challenges (Moschkovich 2013; Prediger 2019).

One explanation for the low number of identified potential linguistic challenges might be that experts named linguistic features that are only considered challenging for students from an expert’s perspective, but not from the perspective of pre-service teachers. However, the participants as a group identified most of the challenges at least once (40 of 44). This indicates that the pre-service teachers’ judgement did largely overlap with the expert ratings, but that they seemed to have difficulties with identifying the potential linguistic challenges reliably.

To gain a clearer view on the potential linguistic challenges that participants *were* able to identify, we distinguished between cross-disciplinary and mathematics-specific potential linguistic challenges as well as between the word level and the sentence level for RQ2, RQ3, and RQ4.

### Pre-Service Teachers’ Subjective Judgement of Disciplinarity

We consider the disciplinarity of academic language to be a subjective judgement. The question if a linguistic feature is cross-disciplinary or mathematics-specific might depend on contextual or individual factors (Uccelli et al. 2015; Zwiers 2014). In RQ2, we investigated how pre-service teachers themselves categorized the potential linguistic challenges regarding disciplinarity and compared it to the experts’ judgement. The results from RQ2 support the ambiguity of this categorization: It seems that the distinction between mathematics-specific and cross-disciplinary language in a mathematical expository text is inconsistent for some potential linguistic challenges, particularly words and phrases that occur both in academic and non-academic contexts, like *allgemeingültig *[universally valid], *kalkulieren *[calculate] or *ist gleich *[is equal to]. For other potential linguistic challenges that are more typical, like *gleichseitig *[equilateral] or complex sentence structures, there was strong agreement among pre-service teachers, and between pre-service teachers and the expert group. This supports the assumption by previous research that the lines between cross-disciplinary and mathematics-specific academic language are often blurred by individual and contextual factors (Zwiers 2014). This further indicates that fostering language-responsive teaching with expository texts requires precision regarding the question of what the target and purpose of language-responsive adaptations is.

Furthermore, with regard to RQ2, the majority of potential linguistic challenges that were categorized by participants as being mathematics-specific (see Fig. 3) were on the word level, which was less pronounced in the experts’ categorization (see Table 3). Cross-disciplinary challenges were distributed more equally between the word and the sentence level, and more similar to the experts’ categorization. This indicates a tendency that academic language is perceived by pre-service teachers to be predominantly an issue of mathematical terms and phrases compared to the expert group, a finding which supports previous research and extends it to expository texts (Jost et al. 2017).

Previous research has shown that students differ in their need for support in mathematics-specific and cross-disciplinary academic language, as students from higher educated or academic households might come into contact with cross-linguistic academic language at home (Snow and Uccelli 2009). Distinguishing and appreciating disciplinarity of potential linguistic challenges is thus a vital aspect for linguistically responsive teaching but, based on our results, there is a lot of ambiguity regarding this subjective categorization.

### Differences in Identifying Challenges by Disciplinarity and Linguistic Level

For RQ 3 and RQ4, the distinction of disciplinarity of the identified linguistic challenges was based on the experts’ subjective judgement, irrespective of the pre-service teachers’ own subjective judgement for RQ2. This was not done with the goal to classify pre-service teachers’ judgements as correct or incorrect. For these analyses, we could also have picked the categorization of the majority of pre-service teachers, or the subjective categorization of each individual. However, to make these results comparable with existing research and consistent with our theoretical background, we used the experts’ categorization, which was arguably based most closely on previous research on subject-specific academic language (e.g., Schleppegrell 2001, 2007, 2010; Snow and Uccelli 2009; Zwiers 2014).

The analyses for RQ3 show that (1) potential linguistic challenges that were categorized by the experts as mathematics-specific were identified more often than cross-disciplinary challenges, (2) potential linguistic challenges were identified more often on the word level than on the sentence level, and (3) the interaction effect indicates, depending on the reading, that a) the tendency towards identifying more linguistic challenges on the word level than on the sentence level is stronger for cross-disciplinary challenges and b) the effect that mathematics-specific potential linguistic challenges are identified more often occurs predominantly on the sentence level.

Regarding (1), pre-service teachers might be more attentive for mathematics-specific linguistic challenges because the text was clearly mathematics-related, and mathematics-specific features might be more salient. However, pre-service teachers were explicitly asked to focus both on mathematics-specific and cross-disciplinary potential linguistic challenges. Alternatively, the linguistic challenges that were categorized by the experts as cross-disciplinary might be harder to identify or considered less challenging by pre-service teachers.

Regarding (2), the potential linguistic challenges at the word level might have been easier for pre-service teachers to identify as they are noticeable on the surface of the text, regardless of sentence structure or content (Wallner 2021). Additionally, pre-service teachers might see lexis as the main challenging aspect of academic language instead of sentence-level language features (Zwiers 2007). Technical terms are often emphasized in mathematics classrooms, making them more apparent and relevant to pre-service teachers (Schütte 2009). However, the interaction effect showed that the main difference between disciplinarity was based on challenges at the sentence level, not just the word level, which partly contradicts the assumption that pre-service teachers consider mathematics-specific linguistics challenges to be primarily on the word level.

Our results indicate that pre-service teachers do not identify all aspects of linguistic challenges in mathematical texts equally often, and that considering disciplinarity and linguistic levels helps to specify these deficits. This adds to existing research (e.g., Jost et al. 2017, Prediger et al. 2019) by highlighting pre-service teachers’ detailled limitations in identifying linguistic challenges in mathematics texts, particularly those on the sentence level which were considered to be cross-disciplinary by experts. Addressing such deficits is in line with Lucas and Villegas (2013) and Prediger (2019) who emphasize the importance of the teachers’ role in incorporating various aspects of language in the classroom. By identifying and understanding linguistic challenges better and more comprehensively, pre-service teachers can better support students in overcoming language-related difficulties, evaluate their performance more adequately, and provide effective support.

### Differences by Pre-Service Teachers’ Subjects

Regarding RQ4, we expected that pre-service teachers of different subjects might identify potential linguistic challenges differently. We assumed that pre-service teachers of German or English language arts might be more sensitive to cross-disciplinary potential linguistic challenges, while mathematics pre-service teachers might be able to identify mathematics-specific challenges more often.

Our results showed no meaningful differences between pre-service teachers of these two subjects in any of the effects. Effect sizes for these analyses were very small, while post-hoc sensitivity analyses indicated that the statistical power would have sufficed to detect effects of meaningful size. This indicates that the nonsignificance was not due to a statistically insufficient design. The lack of evidence for an effect of school subject was surprising, particularly as the comprehension test confirmed that pre-service teachers of mathematics did understand the mathematical content of the expository text better, which indicates that the groups were different with regard to their content knowledge. To investigate to what extent teacher education programs can foster the ability to identify potential linguistic difficulties or if pre-service teachers already differ when choosing their subjects, longitudinal data would be necessary. This could also include data from in-service teachers and provide insights on how the ability develops during their career.

### Implications, Limitations, and Future Directions

The identification of potential linguistic challenges in mathematical texts is considered an important ability for teachers. Based on our results, there is a potential for training pre-service teachers with regard to the ability to identify potential linguistic challenges in expository texts, which could be beneficial for their linguistically responsive teaching. Such a training could address the possible misconception that potential linguistic challenges are found mainly on the word level and specifically improve identification of challenges on the sentence level. Moreover, even at the word level, there is still plenty of room for improvement regarding the amount of identified potential challenges. Previous studies showed that such interventions can be effective for in-service teachers and pre-service teachers (e.g., Kalinowski et al. 2020; Prediger 2019), but further research is needed in order to specifically investigate how to foster the identification of potential linguistic challenges in expository texts, and how pre-service teachers benefit from learning opportunities in this area (see also Brandt et al. 2023).

In the present study, we focused on only one aspect of professional teaching competence regarding linguistically responsive mathematics teaching. Yet, the identification of potential linguistic challenges is only the first step to properly designing and altering expository texts, and it is highly likely that the process includes many iterations of design, analyses, implementation and evaluation. In line with previous research, we assume that the identification of potential linguistic challenges is the prerequisite for many other aspects of linguistically responsive teaching (e.g., Bunch 2013; Fillmore and Snow 2002; Lucas and Villegas 2013; Lucas et al. 2008; Moschkovich 2013; Prediger 2019). Therefore, our study addressed an important first step for further analyses on the implementation of expository texts in linguistically responsive teaching.

Furthermore, individual differences between pre-service teachers might exist that determine which potential linguistic challenges are identified. For example, in our study, there was an unintentional high percentage of female participants. Although there is no evidence of a gender effect in existing research, this might be taken into account when interpreting the results.

There is still relatively little research that looks at mathematical expository texts from a linguistic perspective. In our study, we used a text that focused on one specific geometric theorem for ninth-graders as a first step into this direction. The topic and mathematical subdomain of the text, the genre of expository texts and the age group as well as the individual characteristics of the students reading the text might greatly influence what can be considered a challenging feature of academic language, and how teachers should react to it. Other texts and contexts might provide other, specific challenges and configurations of linguistic features. The heterogeneity of what pre-service teachers and experts consider challenging in the present study illustrates how subjective and situated this assessment can be. Therefore, transferring our approach to other contexts, for example oral discourse, other mathematical subdomains, or a different target group of students, could lead to different results.

## Conclusion

Language plays a key role in mathematics education, making research on teacher competence in this area vital. Our study offers a more nuanced look at the prerequisites for linguistically responsive teaching with mathematical expository texts. Identifying potential linguistic challenges in these texts is a key step in effectively integrating language in mathematics classrooms, and one that has a great potential for improvement among pre-service teachers.

## Figures and Tables

**Fig. A.1:**
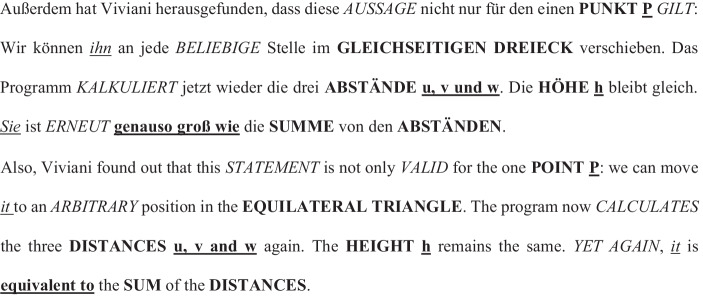
Example paragraph from the identification task with translation (original in German). Potential linguistic challenges on the word level are in capitals, challenges on the text and sentence level are underlined, mathematics-specific challenges are in bold, cross-disciplinary challenges are in italics (no highlighting was used in the original version)

## Appendix

### Appendix B

These sentences were categorized by the experts as potential linguistic challenges because of the complex sentence structure:

1. Angezeigt wird auch die Höhe h.
2. Die Entdeckung von Viviani ist allgemeingültig, weil die Summe von den drei Abständen immer genauso lang ist wie die Länge von der Höhe h, egal wohin der Punkt P verschoben wird
3. Der Satz von Viviani gilt immer, egal wo der Punkt P im gleichseitigen Dreieck ist, weshalb der Satz von Viviani heißt.

## Footnotes

1 The Flesh reading ease score was initially constructed for English texts (Flesch 1948) and adapted for German texts by Amstad (1978). It is calculated as *FREgerman* = 180 − (average sentence length in words) − 58.5 × (average word length in syllables). The value is interpretetd as “sehr schwierig” (< 20), “schwerig” (< 30), “anspruchsvoll” (< 40), “durchschnittlich” (≈ 50), “einfach” (> 60), “leicht” (> 70), and “sehr leicht” (> 80).

2 The expert group was composed of researchers from the ERLE research center and included three professors from language arts, two professors from mathematics education, and four doctoral and postdoctoral researchers. The procedure was non-standardized: The expert group went through the text in separate and joint group meetings, discussed possible challenges, and reached a consensus.

3 Features that were named as potential linguistic challenges by participants but not by the expert group were checked by the coders in order to extent the list of challenges. None of these features were considered to be potential linguistic challenges based on the theoretical and empirical foundations described earlier, thus no further discussion in the expert group was considered necessary. For example, this was the case for “zu wenig verbale Veranschaulichung” [“not enough verbal illustration”] or “Aufzählung der Werte klingt monoton” [“Listing of the values sounds monotonous”].

4 In few instances (3.5%) where participants named the same potential linguistic challenge in both answer fields, it was counted twice for analyses in RQ2, but only once for RQ1, RQ3, and RQ4.

5 To check for performance differences between participants of varying experience, we included the participants’ program (bachelor vs. master) and their semester as control variables. Since both variables did not have a significant influence on the identification of potential linguistic challenges when included in the full model (see 2.4), we did not further include them in the following analyses, *F*(1, 113) = 1.479, *p* = 0.227, part. *η*^*2*^ = 0.01; *F*(1, 113) = 1.859, *p* = 0.175, part. *η*^*2*^ = 0.02.

6 Due to a floor effect, the assumption of normality was violated. Simulations with robust methods using the R package *WRS2 * (Mair and Wilcox 2022) indicated no substantial bias due to this skewness, but *F*-values might be slightly overestimated due to the underestimated standard deviation.
